# Association between type 2 diabetes and autoimmune liver disease: An integrated analysis of Mendelian randomization and clinical samples

**DOI:** 10.1097/MD.0000000000047579

**Published:** 2026-02-06

**Authors:** Luya Wen, Chen’guang Su, Hewen Li, Jian Li, Jinlong Liu

**Affiliations:** a Department of Hepatobiliary Surgery, Affiliated Hospital of Chengde Medical University, Chengde, Hebei Province, China; b Department of Thoracic Surgery, The Second Hospital of Hebei Medical University, Shijiazhuang, Hebei Province, China; c Department of Minimally Invasive Spine Surgery, Affiliated Hospital of Chengde Medical University, Chengde, Hebei Province, China; d Hebei Key Laboratory of Panvascular Diseases, Chengde, Hebei Province, China.

**Keywords:** autoimmune liver disease, causal effect, genome-wide association studies, Mendelian randomization, type 2 diabetes

## Abstract

This study aims to determine the potential causal relationship between type 2 diabetes (T2D) and autoimmune liver disease (AILD) using Mendelian randomization (MR) combined with clinical case analysis. Summary statistics for T2D, autoimmune hepatitis, primary biliary cholangitis (PBC), and primary sclerosing cholangitis (PSC) were sourced from open genome-wide association study databases. The IVW method was used as the primary analysis. Additional sensitivity analysis was also performed to validate our results. Subsequently, clinical information on patients with AILD was collected retrospectively, while multiple potentially confounding independent effects were assessed using multivariate logistic regression analysis. The results of the forward MR analysis showed that genetically predicted T2D was associated with reduced risk of PSC (IVW: odds ratio [OR] = 0.85, 95% confidence interval [CI], 0.77–0.94, *P* = .001). Furthermore, the results of the reverse MR analysis revealed the genetically predicted PBC (OR = 1.96, 95% CI 1.31–3.40, *P* = .016) had a significant correlation with the higher risk of T2D (IVW: OR = 1.02, 95% CI, 1.00–1.04, *P* = .025). An analysis of the clinical sample revealed that the prevalence of T2D among patients with AILD was 27.6%. Notably, multifactorial logistic regression analysis indicated that immunoglobulin G and total bilirubin levels may serve as independent factors influencing the occurrence of T2D. Genetic evidence demonstrated that T2D reduced the risk of PSC, while PBC increased the risk of T2D. Clinical data further confirmed a high prevalence of T2D in patients with autoimmune liver disease, suggesting a bidirectional relationship that warrants further validation.

## 1. Introduction

Autoimmune liver disease (AILD) is a group of chronic, heterogeneous diseases with a poor prognosis that can lead to liver failure or hepatocellular carcinoma. The classical subtypes of AILD include autoimmune hepatitis (AIH), primary biliary cholangitis (PBC), and primary sclerosing cholangitis (PSC).^[1]^ Currently, the pathogenesis of AILD remains unclear. Evidence suggests that the incidence and prevalence of AILD are progressively increasing worldwide.^[2]^ However, treatment regimens based on immunotherapy^[2]^ or ursodeoxycholic acid (UDCA)^[3]^ are not effective in controlling disease progression. Therefore, identifying risk factors for AILD is essential for developing novel therapeutic strategies.

Diabetes mellitus is one of the most prevalent endocrine disorders and among the leading causes of death worldwide.^[4]^ According to epidemiological data from the International Diabetes Federation, the global number of individuals with diabetes mellitus is projected to reach 783 million by 2045, with type 2 diabetes (T2D) accounting for >90% of all cases.^[5]^ T2D is a complex glucose metabolism disorder characterized by insulin resistance and relative insulin deficiency.^[6]^ Increasing evidence indicates that the pathogenesis of T2D is multifactorial,^[7]^ involving interactions between genetic predisposition, lifestyle, and environmental factors.^[8]^

In recent years, several observational studies have highlighted the association between T2D and AILD. A propensity score-matched analysis using data from the UK Biobank showed that AILD was associated with a higher incidence of T2D.^[9]^ It is well known that the liver plays a crucial role in maintaining glucose homeostasis. Immune-mediated liver injury may affect glucose metabolism and insulin secretion.^[10]^ Furthermore, a large observational study reported that T2D is associated with an increased risk of hospitalization or death due to AILD.^[11]^ However, these observational findings may be influenced by various confounding variables, such as obesity^[12]^ and inflammatory bowel disease.^[13]^ In addition, the possibility of reverse causality cannot be excluded. Therefore, it is essential to use more precise research methods to clarify the potential causal relationship between T2D and AILD.

Mendelian randomization (MR) is an epidemiological approach based on genome-wide association study (GWAS) data used to infer potential causal relationships between risk factors and outcomes.^[14]^ Because alleles are randomly assigned at conception, in a manner analogous to randomized controlled trials, MR minimizes the influence of confounding factors and reverse causality.^[15]^ Furthermore, the degree of causal association between T2D and AILD was further validated through the collection and analysis of clinical samples, providing more robust evidence for causal inference. In this study, we aimed to investigate the causal relationship between T2D and AILD using MR analysis combined with clinical data.

## 2. Materials and methods

### 2.1. Study design

**Figure 1.**
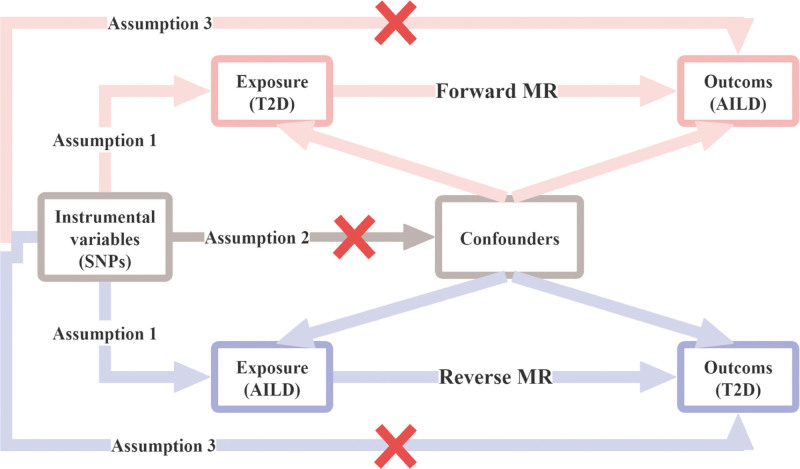
Flowchart of bidirectional Mendelian randomization analysis in this study. AILD = autoimmune liver disease; SNP = single-nucleotide polymorphism; T2D = type 2 diabetes.

The study was designed as a two-sample MR analysis. Specifically, we first defined T2D as the exposure factor to perform a forward MR analysis. Subsequently, AILD was selected as the exposure factor for a reverse MR analysis. It is important to note that all MR analyses must satisfy 3 key assumptions: the chosen instrumental variables (IVs) must be guaranteed to be strongly correlated with the exposure phenotype; IVs must be independent of confounding factors related to exposure phenotype and outcome; and IVs can only affect the outcome through exposure phenotype, rather than through any other pathway.^[16]^ The study design is shown in Figure 1. Meanwhile, our MR analysis was compliant with the STROBE-MR guidelines to bolster the rigor of the study (see Table S1, Supplemental Digital Content, https://links.lww.com/MD/R332).^[17]^

### 2.2. Data sources

All GWAS data for exposures and endings were obtained from the Integrative Epidemiology Unit Open GWAS project (https://gwas.mrcieu.ac.uk/), which is publicly available and does not require specific permissions for access. We screened the IVs for T2D from the recent publication’s GWAS summary statistics, which including 38,841 T2D cases and 451,248 controls of European ancestry (GWAS ID: ebi-a-GCST90018926).^[18]^ The GWAS IDs for AIH (821 AIH cases and 484,413 controls of European ancestry) and PBC (2764 PBC cases and 10,475 controls of European ancestry) were ebi-a-GCST90018785^[18]^ and ebi-a-GCST003129,^[19]^ respectively. The GWAS summary statistics for PSC (GWAS ID: ieu-a-1112)^[20]^ included 2871 PSC cases and 12,019 controls of European ancestry. Since the GWAS summary statistics were all publicly available, we did not require patient consent or informed consent. The details of these datasets in this study are presented in Table 1.

**Table 1:** Characteristics of the dataset used for Mendelian randomization analysis.

Item	GWAS ID	PMID	Author	Year	n case	n control	n sample size	Population	n SNPs
T2D	ebi-a-GCST90018926	European Bioinformatics Institute	Saori S, et al.	2021	38,841	451,248	490,089	European	24,167,560
AIH	ebi-a-GCST90018785	European Bioinformatics Institute	Saori S, et al.	2021	821	484,413	485,234	European	24,198,482
PBC	ebi-a-GCST003129	European Bioinformatics Institute	Heather JC, et al.	2015	2764	10,475	13,239	European	1,124,241
PSC	ieu-a-1112	Integrative Epidemiology Unit	Sun GJ, et al.	2017	2871	12,019	14,890	European	7,891,603

AIH = autoimmune hepatitis, GWAS = genome-wide association study, PBC = primary biliary cholangitis, PSC = primary sclerosing cholangitis, SNPs = single nucleotide polymorphisms, T2D = type 2 diabetes.

### 2.3. IV selection

We set strict quality control for the selection of IVs based on the 3 key hypotheses outlined by Bownden et al.^[21]^ For the first hypothesis, the single nucleotide polymorphisms (SNPs) closely associated with exposure variables were screened according to the *P* < 5 × 10^‐8^ criterion. Due to the low number of AIH-associated IVs screened and to better comply with the random control principle, we used a more lenient threshold (*P* < 5 × 10^‐6^) in our study based on previous experience.^[22,23]^ Subsequently, to keep SNPs independent, we performed linkage disequilibrium analysis (*r*^2^ > 0.001; clumping window < 10,000 kb) based on the European 1000 Genomes Project reference panel. In addition, by calculating the *F*-statistic, we determined the probability that each SNP was a weak instrument. Based on previous experience, when *F*-statistic >10, the genetic variation was deemed a weak IV, which may have caused bias into the research results.^[24]^ The specific equation for the *F*-statistic is defined as: *F* = β^2^/SE^2,[25]^ where β represents the effect size on exposure, and SE signifies the estimated standard error of SNP on exposure. For the second condition, we used the PhenoScanner tool^[26]^ to examine each SNP individually for associations with other known confounders,^[27,28]^ applying a threshold of *P* < 1 × 10^‐5^. Any SNPs found to be associated with these confounders were excluded. For the third hypothesis, we examined the correlation between the screened SNPs and the outcome on a case-by-case basis to ensure that *p*-exposure > *p*-outcome. Finally, to ensure consistent effect alleles in the exposure and outcome datasets, SNPs were harmonized to exclude those with palindromic alleles SNP and incompatible alleles SNP.^[29]^ We did not perform proxy SNPs substitutions in order to ensure the quality of the IV screening.

### 2.4. MR analysis

The inverse-variance weighted (IVW) method was applied as the main method of MR analysis to make a rigorous determination of the association between exposure and outcome. The main design idea of IVW is that, first, the Wald ratio is estimated individually for each SNP. Subsequently, a pooled effect size is obtained by aggregating the Wald ratios of all SNPs. Since its calculation process does not take into account the presence of an intercept term, IVW has the most accurate statistical results when there is no horizontal pleiotropy.^[30]^ In addition, we employed MR-Egger regression, weighted median method, simple mode, and weighted mode methods as additional analytical methods. When horizontal pleiotropy exists, MR-Egger regression can optimize the IVW estimate by modulating the intercept and regression slope.^[21]^ The weighted median method allows the results to remain stable when no >50% of the IVs are invalid.^[31]^ Finally, weighted mode method and simple mode method were used to further evaluate the credibility of IVW results.^[32]^ To confirm the robustness and reliability of the results, we performed a sensitivity analysis of the MR analysis. Specifically, potential heterogeneity was detected by Cochran *Q* statistic.^[33]^ Where *P* > .05 indicates that there is no potential heterogeneity in the results, which was calculated using a fixed effects IVW model; otherwise, a random effects IVW model was chosen.^[34]^ Finally, the heterogeneity results were visualized by plotting a funnel plot. Furthermore, we used MR Egger regression to identify potential horizontal pleiotropy. The *P*-value of the MR-Egger intercept >0.05 indicates that there is no horizontal pleiotropy.^[35]^ Finally, we assessed the impact of single SNPs on the overall results.^[36]^

### 2.5. Study population

We independently conducted a retrospective data collection of patients diagnosed with AILD, including AIH, PBC, and PSC, to validate the findings of the reverse MR analysis. These patients were admitted to the Affiliated Hospital of Chengde Medical University between August 2016 and July 2024. The inclusion criteria were as follows: a diagnosis of AILD based on abdominal imaging (such as abdominal ultrasound and cholangiography), clinical signs of cholestasis, and biochemical evidence; positive results from immunological assays for AILD-related autoantibodies; and regular treatment with UDCA following diagnosis. The exclusion criteria were as follows: patients with advanced liver complications at presentation, such as hepatocellular carcinoma or a history of liver transplantation; patients with severe extrahepatic autoimmune disorders (e.g., systemic lupus erythematosus, rheumatoid arthritis) that required long-term immunosuppressive therapy which could confound the assessment; patients who were pregnant or lactating; patients with a history of type 1 diabetes or gestational diabetes; and patients who were seen only in the outpatient setting, leading to incomplete or missing essential diagnostic test data.

The diagnosis of AILD was made in accordance with the International Classification of Diseases, 10th Revision. T2DM was defined as a fasting blood sugar of ≥126 mg/dL or a random blood sugar of ≥200 mg/dL, a glycosylated hemoglobin level of ≥6.5%. Patients who were missing diagnostic information but were using medications related to T2D or insulin for glycemic management also met the criteria. Regrettably, patients with PSC were excluded from the study due to the small sample size (n = 6), which was insufficient for stratifying subgroups by sex and age. Ultimately, 38 AIH patients and 49 PBC were included in the study. The extracted patient case information was rendered innominate prior to further collation.

### 2.6. Baseline data and study variables

Information on demographic and clinical characteristics (including gender, age, child–Pugh classification, BMI, immunologic features, liver function, hematologic parameters, and relevant comorbidities) was extracted from the electronic health record system. In addition, noncollinear variables were selected as covariates in the logistic regression analyses, including gender, age, Child–Pugh classification, BMI, immunoglobulin G (IgG), platelet count, alanine aminotransferase, bilirubin, and albumin. For the subgroup analyses, all continuous variables in the logistic regression models were converted into dichotomous variables based on the threshold values indicated in the laboratory reports.

### 2.7. Statistical analysis

MR analysis was performed using the “TwoSampleMR” package (version 0.5.8) with R (version 4.2.3), where results were reported as odds ratios (OR) with their corresponding 95% confidence intervals (CIs). SPSS 25.0 software (IBM, Armonk) was utilized to analyze the clinical data. Continuous variables that follow a normal distribution are presented as mean ± standard deviation, and continuous while with a skewed distribution are expressed as medians (interquartile ranges). Group differences were analyzed using Student *t* test or Mann–Whitney test. Categorical variables were described as percentages and group differences were assessed using the Chi-square test (χ^2^) or Fisher exact test. Logistic regression analysis was reported as OR along with their corresponding 95% CI. In this case, covariates with a *P*-value of <0.1 in the univariate logistic regression analysis will be included in the multivariate logistic regression analysis, adjusting for all covariates. A significance level of a two-sided *P*-value <.05 was used for all statistical tests.

## 3. Results

### 3.1. Multiple SNPs strongly associated with T2D and AILD subtypes were retained as valid instruments

SNPs strongly associated with exposure were screened according to the screening criteria described above. Then, palindromic alleles and incompatible alleles were excluded after harmonization of exposure and outcome. Finally, there were 177 SNPs associated with T2D and AIH, 85 SNPs associated with T2D and PBC, 139 SNPs associated with T2D and PSC, 13 SNPs associated with AIH and T2D, 25 SNPs associated with PBC and T2D, and 18 SNPs associated with PSC and T2D that were identified, respectively. The *F*-values for each of the SNP is >10, indicating that there was no weak IV. The specific information on SNPs was provided in Tables S2–S7, Supplemental Digital Content, https://links.lww.com/MD/R333.

### 3.2. Forward MR analysis of the effects of T2D on AILD

**Figure 2.**
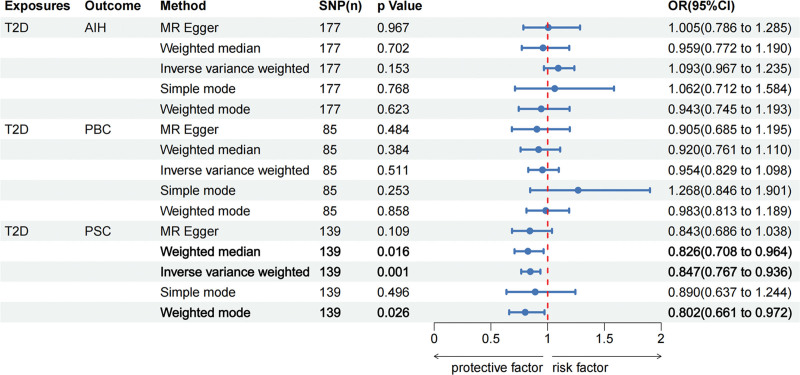
The forward Mendelian randomization analysis results of T2D and autoimmune liver disease (AIH, PBC, and PSC). AIH = autoimmune hepatitis; PBC = primary biliary cholangitis; PSC = primary sclerosing cholangitis; T2D = type 2 diabetes.

**Figure 3.**
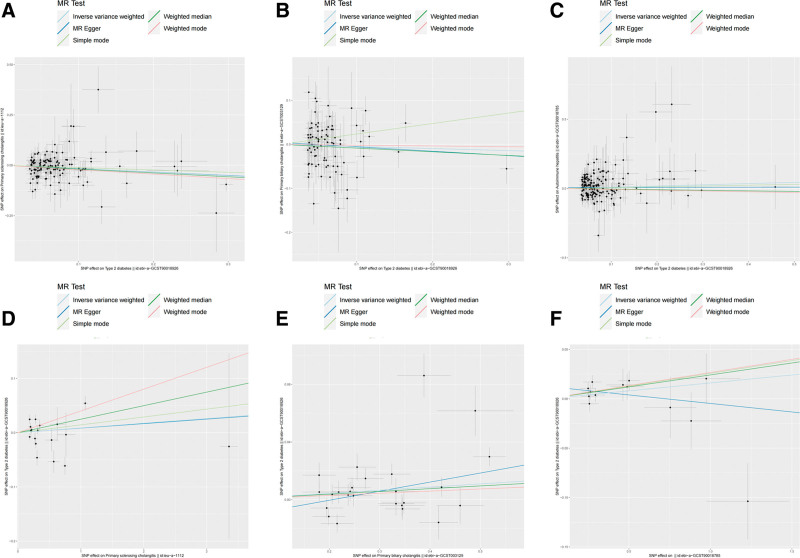
The scatter plot of bidirectional Mendelian randomization analysis. (A) T2D on AIH; (B) T2D on PBC; (C) T2D on PSC; (D) AIH on T2D; (E) PBC on T2D; and (F) PSC on T2D. AIH = autoimmune hepatitis; PBC = primary biliary cholangitis; PSC = primary sclerosing cholangitis; T2D = type 2 diabetes.

Based on the IVW method estimates, genetically predicted T2D was associated with a lower risk of PSC (OR = 0.85, 95% CI, 0.77–0.94, *P* = .001), and this result was supported by weighted median (OR = 0.83, 95% CI, 0.71–0.96, *P* = .016) and weighted mode (OR = 0.80, 95% CI, 0.66–0.97, *P* = .026). However, there was no evidence for a direct causal effect of T2D on AIH (IVW: OR = 1.09, 95% CI, 0.97–1.24, *P* = .153) and PBC (IVW: OR = 0.95, 95% CI, 0.83–1.10, *P* = .511), and these results were supported by all additional methods. The results of the MR analysis can be found in Figures 2 and 3A–C.

Subsequently, sensitivity analyses were performed to verify the robustness of the results (Table 2). The results of Cochran *Q* test showed no heterogeneity in the results (T2D–AIH, *P* = .479; T2D–PSC, *P* = .088). Although heterogeneity was observed between T2D and PBC (*P* < .001), this is acceptable as the study was analyzed primarily using a multiplicative random effects model. At the same time, we applied funnel plots to visualize the results of heterogeneity (Fig. S1A–C, Supplemental Digital Content, https://links.lww.com/MD/R332). In addition, the data from the Egger regression tests showed that no horizontal pleiotropy was observed for any of the results (T2D–AIH, *P* = .443; T2D–PBC, *P* = .668; T2D–PSC, *P* = .963), indicating that the results were not affected by potential confounders. The leave-one-out sensitivity analysis showed that the causal relationship between T2D and AILD was not affected by any single SNP (Fig. S2, Supplemental Digital Content, https://links.lww.com/MD/R332). The forest plots (Fig. S3, Supplemental Digital Content, https://links.lww.com/MD/R332) display causal estimates for each SNP, showing that T2D reduces PSC risk, consistent with our main findings. In summary, the results of the sensitivity analysis are consistent with our main findings, and therefore our results are robust.

**Table 2:** Sensitivity analysis of the forward Mendelian randomization analysis results of type 2 diabetes on autoimmune liver disease.

Exposure	Outcome	Method	nSNP	Cochran *Q* test			Intercept term			
				Q	Q_df	*P*	Egger_intercept		SE	*P*
T2D	AIH	MR Egger	177	175.708	175	.471	0.007	0.009		.443
		Inverse variance weighted	177	176.303	176	.479				
T2D	PBC	MR Egger	85	138.128	83	<.001	0.004	0.010		.668
		Inverse variance weighted	85	138.436	84	<.001				
T2D	PSC	MR Egger	139	160.993	137	.079	<0.001	0.008		.963
		Inverse variance weighted	139	160.996	138	.088				

AIH = autoimmune hepatitis, MR = Mendelian randomization, PBC = primary biliary cholangitis, PSC = primary sclerosing cholangitis, SE = standard error, T2D = type 2 diabetes.

### 3.3. Reverse MR analysis of the effects of AILD on T2D

**Figure 4.**
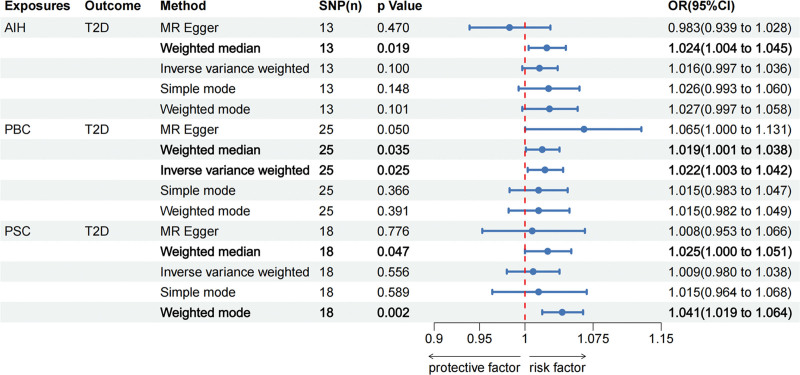
The reverse Mendelian randomization analysis results of autoimmune liver disease (AIH, PBC, and PSC) and T2D. AIH = autoimmune hepatitis; PBC = primary biliary cholangitis; PSC = primary sclerosing cholangitis; T2D = type 2 diabetes.

To complete the directionality of the causal relationship between T2D and AILD, we also performed a reverse MR analysis. The IVW analysis results revealed that genetic prediction PBC had a significant correlation with the higher risk of T2D (OR = 1.02, 95% CI, 1.00–1.04, *P* = .025), and weighted median also showed similar results (OR = 1.02, 95% CI, 1.00–1.04, *P* = .035). However, the causal relationship between other types of AILD and T2D was insignificant, including AIH (IVW: OR = 1.09, 95% CI, 0.97–1.24, *P* = .153) and PSC (IVW: OR = 1.09, 95% CI, 0.97–1.24, *P* = .153). Detailed information of reverse MR analysis was presented in Figures 4 and 3D–F.

Cochran *Q* test revealed that there was no significant heterogeneity between AIH and T2D, and a fixed effects IVW model was used. However, significant heterogeneity was detected in the remaining results (PBC–T2D, *P* < .001; PSC–T2D, *P* < .001). Therefore, in order to estimate the MR effect size, the random effect model was applied (Table 3). We applied funnel plots to visualize the results of heterogeneity, as detailed in Figure S1D–F, Supplemental Digital Content, https://links.lww.com/MD/R332. Additionally, Egger regression tests did not detect any evidence of horizontal pleiotropy. The leave-one-out analysis indicated that omitting any individual SNP does not significantly influence the overall estimate (Fig. S4, Supplemental Digital Content, https://links.lww.com/MD/R332). The forest plots (Fig. S5, Supplemental Digital Content, https://links.lww.com/MD/R332) reveal a causal effect of PBC in increasing the risk of T2D, as estimated by individual SNPs. Overall, the results of the sensitivity analyses support our primary findings. Despite the challenges posed by heterogeneity, this is acceptable due to the use of a random effects model. Forest plots are presented in Figure S5, Supplemental Digital Content, https://links.lww.com/MD/R332.

**Table 3:** Sensitivity analysis of the reverse Mendelian randomization analysis results of autoimmune liver disease on type 2 diabetes.

Exposure	Outcome	Method	nSNP	Cochran *Q* test			Intercept term			
				*Q*	*Q*_df	*P*	Egger_intercept		SE	*P*
AIH	T2D	MR Egger	13	16.047	11	.139	0.013	0.008		.143
		Inverse variance weighted	13	19.683	12	.073				
PBC	T2D	MR Egger	25	79.662	23	<.001	‐0.013	0.009		.170
		Inverse variance weighted	25	86.620	24	<.001				
PSC	T2D	MR Egger	18	75.747	16	<.001	<0.001	0.010		.987
		Inverse variance weighted	18	75.748	17	<.001				

AIH = autoimmune hepatitis, MR = Mendelian randomization, PBC = primary biliary cholangitis, PSC = primary sclerosing cholangitis, T2D = type 2 diabetes.

### 3.4. Clinical validation of distinct T2D risk factors in AILD subtypes

Data from the clinical sample indicated that a total of 24 out of 87 AILD patients (27.6%) had T2D. Among these, 9 AIH patients (23.7%) and 15 PBC patients (30.6%) were diagnosed with T2D. Subsequently, we categorized patients with each subtype of AILD based on the presence or absence of comorbid T2D and analyzed their baseline data (Table 4). As detailed in Table 4, AIH patients with comorbid T2D had a significantly higher prevalence of hypertension than their nondiabetic counterparts (66.7% vs 20.7%; *P* = .016 by χ^2^ test). Furthermore, among patients with PBC, those with T2D exhibited higher IgG test values (*P* = .018) and lower total bilirubin levels (*P* = .046) than patients without T2D. These differences were statistically significant. Multifactorial logistic regression analysis revealed that elevated IgG levels were an independent risk factor for the development of T2D in patients with PBC (OR = 11.30, 95% CI, 1.02–124.89, *P* = .048). Additionally, high total bilirubin levels were identified as a protective factor that reduced the likelihood of developing T2D in patients with PBC (OR = 0.02, 95% CI, 0.001–0.33, *P* = .006). Unfortunately, no independent influencing factors for the occurrence of combined T2D in patients with AIH were identified (Table 5).

**Table 4:** Baselines characteristics of clinical samples.

Factors	AIH			PBC		
	With T2D (n = 9)	Without T2D (n = 29)	*P*	With T2D (n = 15)	Without T2D (n = 34)	*P*
Gender						
Male (n [%])	1 (11.1%)	3 (10.3%)	0.340	2 (13.3%)	2 (5.9%)	.755
Female (n [%])	8 (88.9%)	26 (89.7%)		13 (86.7%)	32 (94.1%)	
Age (mean ± SD, years)	59.2 ± 10.7	57.6 ± 9.6	0.666	62.5 ± 10.3	58.7 ± 9.7	.221
Child-Pugh						
A (n [%])	8 (88.9%)	24 (82.8%)	1.000	12 (80.0%)	24 (70.6%)	.736
B/C (n [%])	1 (11.1%)	5 (17.2%)		3 (20.0%)	10 (29.4%)	
BMI (mean ± SD, kg/m^2^)	22.1 ± 6.0	23.3 ± 3.5	0.467	22.0 ± 4.1	22.3 ± 3.4	.766
Immunologic features						
IgG (mean ± SD, g/L)	16.7 ± 5.2	18.3 ± 6.6	0.462	22.0 ± 5.6	17.3 ± 6.3	.018
ANA (n [%])	6 (66.7%)	24 (82.8%)	0.327	12/15 (80.0%)	29/34 (85.3%)	.966
Liver function						
ALT (median [IQR], IU/L)	20.4 (16.3–34.9)	29.4 (21.0–136.9)	0.107	42.0 (21.5–95.7)	68.7 (30.4–143.5)	.204
AST (median [IQR], IU/L)	32.5 (20.3–87.8)	41.0 (27.0–115.6)	0.420	49.0 (28.0–78.9)	105.6 (36.5–166.4)	.097
ALP (median [IQR], IU/L)	109.0 (80.3–286.0)	163.0 (105.9–234.1)	0.559	165.6 (126.0–194.0)	180.5 (100.8–256.0)	.778
Bilirubin (median [IQR], g/L)	12.3 (8.9–19.0)	10.9 (8.9–16.4)	0.986	18.6 (12.3–36.7)	41.4 (21.8–110.3)	.046
Albumin (median [IQR], g/L)	41.9 (36.6–44.3)	41.2 (35.4–43.3)	0.643	35.1 ± 7.6	36.4 ± 6.1	.532
Blood features						
INR (median [IQR], IU/L)	1.0 (0.9–1.1)	1.0 (1.0–1.1)	0.439	1.1 (1.0–1.3)	1.0 (1.0–1.1)	.397
Platelets (mean ± SD, 10^9^/L)	235.9 ± 100.6	194.3 ± 94.7	0.292	90.0 (67.0–232.0)	147.0 (96.5–211.3)	.380
Comorbidities						
Hypertension (n [%])	6 (66.7%)	6 (20.7%)	0.016	4/15 (26.7%)	12/34 (35.3%)	.553
Hyperlipidemia (n [%])	1 (11.1%)	2 (6.9%)	1.000	1/15 (6.7%)	5/34 (14.7%)	.750

AIH = autoimmune hepatitis, ALP = alkaline phosphatase, ALT = alanine aminotransferase, ANA = antinuclear antibody, AST = aspartate aminotransferase, BMI = body mass index, IgG = immunoglobulin G, INR = international normalized ratio, IQR = interquartile range, PBC = primary biliary cholangitis, T2D = type 2 diabetes.

**Table 5:** Logistic regression analysis of the predictive factors for T2D in AILD.

Factors	AIH (n = 38)				PBC (n = 49)			
	Univariate analysis OR (95% CI)	*P*	Multivariate analysis OR (95% CI)	*P*	Univariate analysis OR (95% CI)	*P*	Multivariate analysis OR (95% CI)	*P*
Gender (male/female)	4.33 (0.30–63.30)	.284			2.46 (0.31–19.38)	.392		
Age (yr, ≥55/<55)	2.14 (0.38–12.20)	.392			3.11 (0.60–16.24)	.179		
Child-Pugh A	1.67 (0.17–16.48)	.662			1.67 (0.39–7.21)	.494		
BMI (kg/m^2^, ≥24/<24)	0.21 (0.02–1.87)	.159			1.01 (0.26–3.99)	.989		
IgG (mg/dL, ≥16/<16)	0.49 (0.11–2.22)	.354			5.13 (1.00–26.33)	.050	11.30 (1.02–124.89)	.048
Platelets (109/L, ≥125/<125)	2.09 (0.22–20.09)	.524			0.41 (0.12–1.43)	.163		
Liver function								
ALT (IU/L, ≥40/<40)	0.15 (0.02–1.39)	.096	0.15 (0.01–2.68)	.195	0.55 (0.16–1.90)	.341		
Bilirubin (mg/mL, ≥21/<21)	0.28 (0.05–4.61)	.524			0.15 (0.04–0.61)	.008	0.02 (0.001–0.33)	.006
Albumin (g/L, ≥35/<35)	1.11 (0.19–6.65)	.906			0.53 (0.15–1.81)	.308		

AIH = autoimmune hepatitis, AILD = autoimmune liver disease, ALT = alanine aminotransferase, IgG = immunoglobulin G, OR = odds ratio, PBC = primary biliary cholangitis, T2D = type 2 diabetes.

## 4. Discussion

To the best of our knowledge, this study is the first MR analysis to investigate the causal relationship between T2D and AILD. Our results demonstrated that T2D was associated with a reduced risk of PSC. In addition, there was a positive genetic association between PBC and an increased risk of T2D. However, no potential causal relationships were observed between T2D and AIH, T2D and PBC, AIH and T2D, or PSC and T2D. Importantly, multiple sensitivity analyses were conducted to confirm the robustness of these findings. Furthermore, based on real-world clinical data from patients with AILD, we identified a higher prevalence of T2D and suggested that IgG and total bilirubin levels may independently influence the development of T2D in patients with PBC.

Previous studies have concluded that T2D is a risk factor for PSC. The results of a national retrospective cohort study conducted by United Kingdom academics showed that T2D increased the risk of hospital admission or death for all common chronic liver diseases, including AILD, compared to the nondiabetic population.^[11]^ However, this retrospective study was influenced by sex and socio-economic status. An insightful animal study also suggests that a high-fat and high-fructose dietary pattern can lead to pathogenic immune autoreactivity in the liver.^[37]^ It is of paramount importance to acknowledge that the high-fat and high-fructose dietary pattern contributes to a T2D-like syndrome in C57BL/6 mice. However, the results of these studies are not consistent with the results of our MR analysis, and the reasons for this phenomenon may include the following. First, the role of confounding factors may be a persistent problem in distorting the findings of observational studies, such as include blood lipid,^[38]^ coronary artery disease,^[39]^ and thyroid disease.^[40,41]^ A cross-sectional study showed that patients with T2D had a significantly higher rate of subclinical hypothyroidism than those without T2D.^[42]^ In addition, the reason that T2D is associated with a reduced risk of PSC may be related to the medications taken by patients. Pioglitazone, a peroxisome proliferator-activated receptor γ agonist, is widely used in hypoglycemic therapy for patients with T2D.^[43]^ Recent studies have shown optimistic outcomes for pioglitazone in the treatment of autoimmune diseases.^[44,45]^ Similarly, Ye et al investigated the therapeutic role of metformin in an Abcb4^‐/‐^ model resembling sclerosing cholangitis.^[46]^ It was revealed that metformin ameliorated liver fibrosis in the cholestatic Abcb4^‐/‐^ model by targeting yes-associated protein inhibition. In summary, treatment modalities and drug choices for patients with T2D may potentially contribute to the reduced risk of developing PSC with T2D. However, the association between T2D with AIH and PBC remains unexplained, limited by the small number of studies and unclear pathway mechanisms.

In a reverse MR analysis, we revealed the possibility that PBC increases the risk of developing T2D. Previous observational trials have shown that PBC may be a potential risk factor for T2D, and these findings are consistent with our study conclusions. Lin et al conducted a 30-year cohort study to provide comprehensive statistics on the incidence of extrahepatic events in patients with PBC.^[47]^ The results showed that the cumulative incidence of T2D in patients with PBC was as high as 30.6%. This was despite the fact that all patients were routinely treated with ursodeoxycholic acid. In addition, an observational study of untreated patients with serum antimitochondrial antibody-positive PBC demonstrated a significant relationship between PBC and high T2D incidence.^[48]^ In 2023, Jensen et al conducted a large meta-analysis and the article summarized data for 77 studies that met the screening criteria^[9]^ and showed that the combined prevalence of PBC in T2D was 18.1% (*I*^2^ = 89%). The meta-analysis also pooled 3 double-arm clinical trials and showed that individuals diagnosed with PBC experience an 80% higher risk of T2D compared to controls.

Our single-arm clinical trial demonstrated that the prevalence of T2D in patients with AILD was 27.6%. Specifically, the prevalence of T2D was found to be 23.7% in patients diagnosed with AIH and 30.6% in those with PBC. These figures are significantly higher than the 9.7% to 18.7% prevalence rates reported in recent epidemiological studies of T2D in China.^[49–51]^ Furthermore, these findings provide additional support for the results of MR analysis that incorporate a genetic framework. To bridge the gap between this genetic association and the underlying biology, we investigated potential mediators using our clinical data. Our multivariate logistic regression identified that elevated IgG levels independently increase the risk of T2D in PBC patients. IgG has been demonstrated to play a significant role in the development of PBC, particularly concerning its glycosylation status.^[52]^ Meng et al confirmed the potential association between IgG N-glycosylation and T2D in patients using ultra-performance liquid chromatography and mass spectrometry.^[53]^ Given that IgG glycosylation is implicated in both PBC pathogenesis and T2D development, it is plausible that the altered immune state in PBC, reflected by specific IgG profiles, may directly contribute to impaired glucose metabolism. Together with our clinical data, the detection of IgG levels in PBC patients is valuable for the early identification of T2D risk. Interestingly, the clinical data also confirmed that elevated levels of total bilirubin may serve as a protective factor in patients with T2D. Previous studies have demonstrated that serum bilirubin functions as a lipid-solubleantioxidant, helping to mitigate the development of insulin resistance by preventing oxidative stress and reducing inflammation.^[54]^ This, in turn, may slow the progression of T2D. These clinical findings offer tangible mechanistic clues, suggesting that the causal pathway from PBC to T2D may be mediated through dysregulated immune activity and perturbations in bile acid metabolism that affect both bilirubin levels and glucose homeostasis. However, larger real-world studies are necessary, as the current research is limited by an insufficient sample size. In summary, this consistency between genetic causality and clinical epidemiology forms a compelling evidence chain, suggesting that the link between PBC and T2D is not merely associative but likely causal.

Regrettably, the molecular mechanisms underlying the causal link between PBC and T2D still cannot be fully revealed. The liver is key in regulating glucose homeostasis, which is disrupted in vivo when immunogenic liver injury occurs.^[55]^ At the same time, activated immune factors increase collagen-I levels through hepatic stellate cells autophagy,^[56]^ which promotes hepatic fibrosis and thus triggers insulin resistance.^[57]^ Accelerated the occurrence and development of T2D. Coincidentally, in 2019, a Belgian case report reported that the application of desiccated ursodeoxycholic acid significantly alleviated the level of hepatic fibrosis in patients with PBC and simultaneously improved insulin resistance.^[58]^ In our clinical cohort, elevated serum IgG levels were identified as an independent risk factor for T2D among patients with AILD, supporting the notion that chronic immune activation and hypergammaglobulinemia in PBC may exacerbate systemic insulin resistance. In addition, it has been established that bile acids play an important role in glucose homeostasis.^[59,60]^ Intrahepatic cholestasis is a characteristic manifestation of PBC and may result in a severe disorder of bile acid circulation.^[61]^ Stagnant bile acids disrupt glucose metabolism by mediating multiple signaling pathways,^[62–64]^ thereby increasing the risk of developing T2D. Integrated insights from genetics, clinical studies, and mechanistic insights suggest that immune dysregulation, hepatic fibrosis, and bile acid imbalance may jointly mediate the bidirectional interaction between PBC and T2D. However, further mechanistic and translational studies are warranted to validate these hypotheses.

Our study possesses several highlights. First, the MR analysis is based on a large-scale, updated GWAS database to obtain IVs, so the results have high confidence and accuracy. Second, MR analyses are better able to avoid potential confounding effects on results compared to traditional observational studies. From a genetic perspective, MR analysis leverages the random distribution of genetic variants within the population, thereby reducing the influence of various uncertain confounders. In terms of the analytical methodology, bias control is maximized by selecting IVs that are strongly correlated with exposure factors while remaining relatively independent of confounders. Furthermore, the application of Egger regression to detect horizontal pleiotropy allows for a deeper investigation into the extent to which potential confounding may impact the results. Not only that, our study more comprehensively explored the causal relationship between the 3 subtypes of AILD and T2D compared to the MR analyses carried out by Lin et al^[65]^ and Lv et al^[66]^ for PBC only. Furthermore, these studies lacked validation with a clinical sample. In contrast, we further argued the results of the MR analysis with additional clinical evidence, enhancing the credibility of the results.

However, a full discussion of the limitations of this study is necessary. First, we evaluated the results using strict thresholds and other test hypotheses, which may produce falsely positive results. Second, because the GWAS data did not provide detailed information on patients with each disease, a more detailed subgroup analysis could not be performed to elucidate the specificity of the results. Third, the genetic background of this study exclusively comprised individuals of European descent. Therefore, the generalizability of our findings to populations worldwide might be limited. Additionally, our clinical data were constrained by the low prevalence of the disease and the single-center design of the study, which results in a relatively small sample size and a lack of control groups. Finally, this clinical study did not validate a forward MR analysis, which requires further investigation in larger prospective studies.

## 5. Conclusions

Our MR analysis results support that genetically predicted T2D has a causal effect on the risk of PSC. Similarly, the results of the reverse MR analysis revealed a causal association between PBC and T2D, supported by data from clinical samples. In addition, we identified the potential role of IgG and total bilirubin as independent influencing factors in patients with PBC combined with T2D, provided new insights into the clinical practice for patients with T2D and AILD.

## Acknowledgments

We thank the above databases for making their data publicly available.

## Author contributions

**Data curation:** Luya Wen.

**Formal analysis:** Chen’guang Su, Hewen Li.

**Methodology:** Chen’guang Su.

**Software:** Hewen Li, Jian Li.

**Validation:** Luya Wen, Jinlong Liu.

**Writing – original draft:** Luya Wen.

**Writing – review & editing:** Chen’guang Su, Hewen Li, Jian Li, Jinlong Liu.

## Supplementary Material




