# Supplementary material for: Association between type 2 diabetes and autoimmune liver disease: An integrated analysis of Mendelian randomization and clinical samples
Source: Medicine (Baltimore). 2026 Feb 6;105(6):e47579. doi: 10.1097/MD.0000000000047579 (PMC12885690; doi:10.1097/MD.0000000000047579)
Supplement: Supplementary file 1 [file medi-105-e47579-s001.docx]

**Figure S1.** Leave-one-out sensitivity analysis of bidirectional Mendelian randomization analysis. **(A)** T2D on AIH; **(B)** T2D on PBC; **(C)** T2D on PSC; **(D)** AIH on T2D; **(E)** PBC on T2D; and **(F)** PSC on T2D. T2D, type 2 diabetes; AIH, autoimmune hepatitis; PBC, primary biliary cholangitis; PSC, primary sclerosing cholangitis.


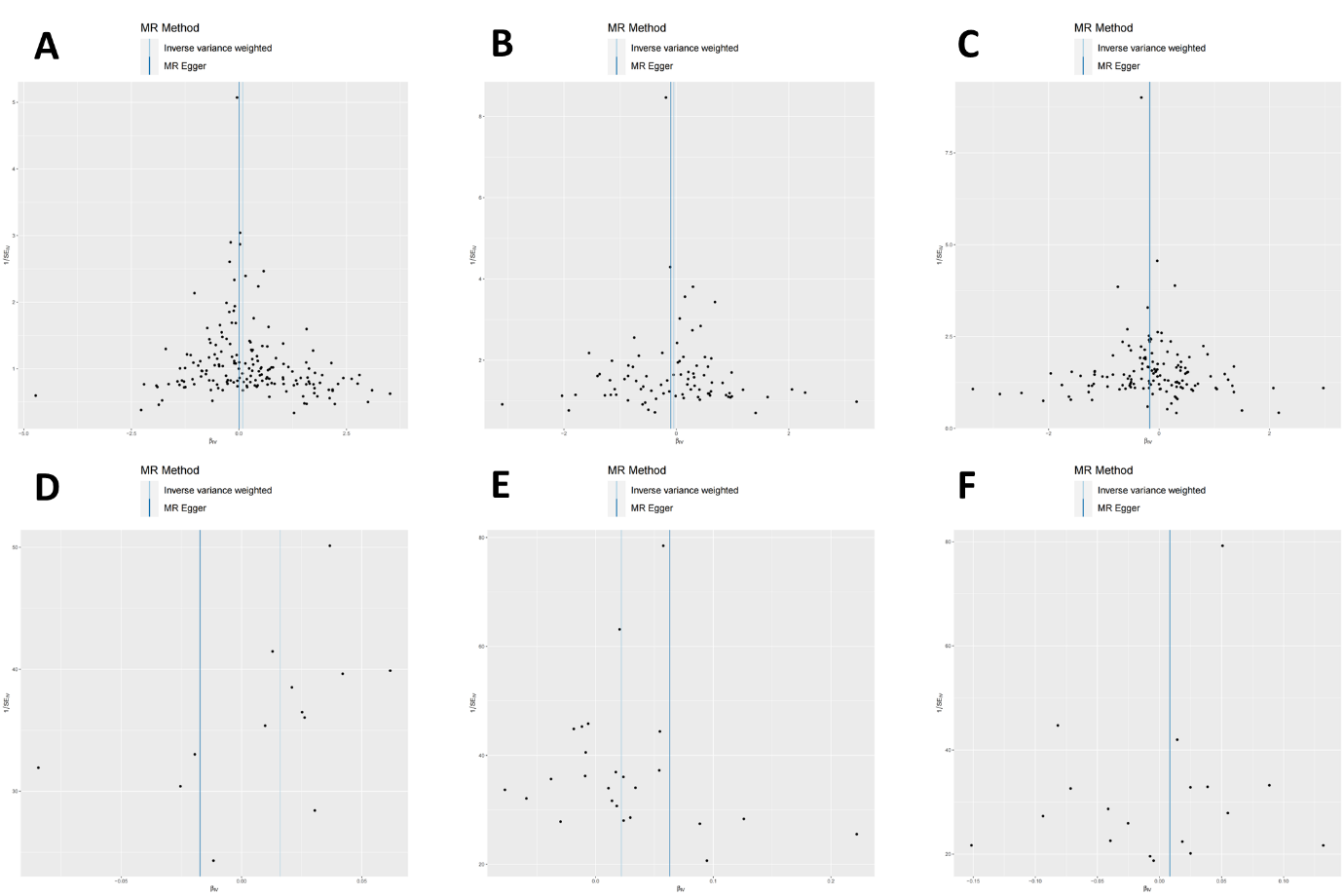


**Figure S2.** The funnel plot of forward Mendelian randomization analysis. **(A)** T2D on AIH; **(B)** T2D on PBC; **(C)** T2D on PSC. T2D, type 2 diabetes; AIH, autoimmune hepatitis; PBC, primary biliary cholangitis; PSC, primary sclerosing cholangitis.


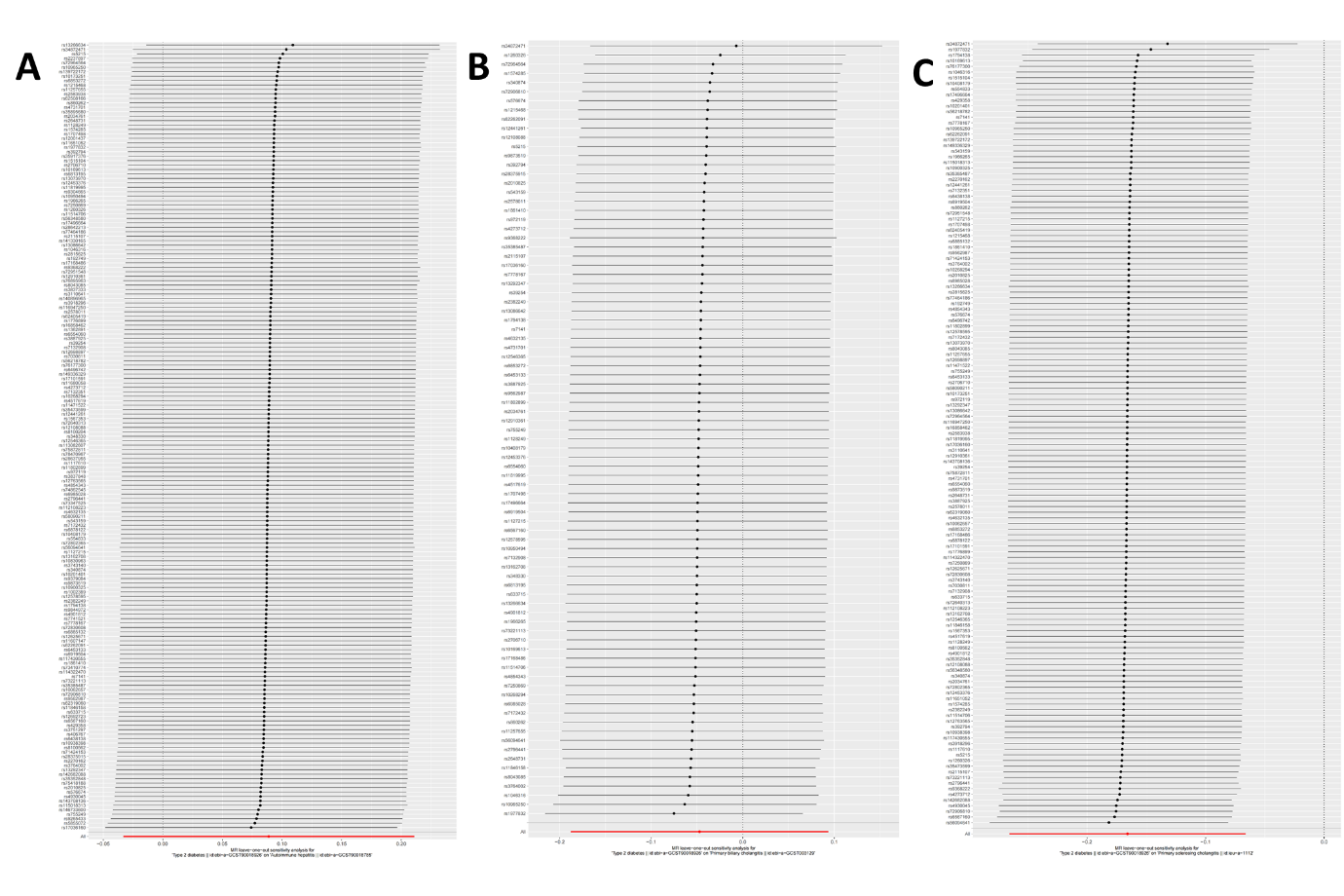


**Figure S3.** The forest plot of forward Mendelian randomization analysis. **(A)** AIH on T2D; **(B)** PBC on T2D; and **(C)** PSC on T2D. AIH, autoimmune hepatitis; PBC, primary biliary cholangitis; PSC, primary sclerosing cholangitis; T2D, type 2 diabetes.


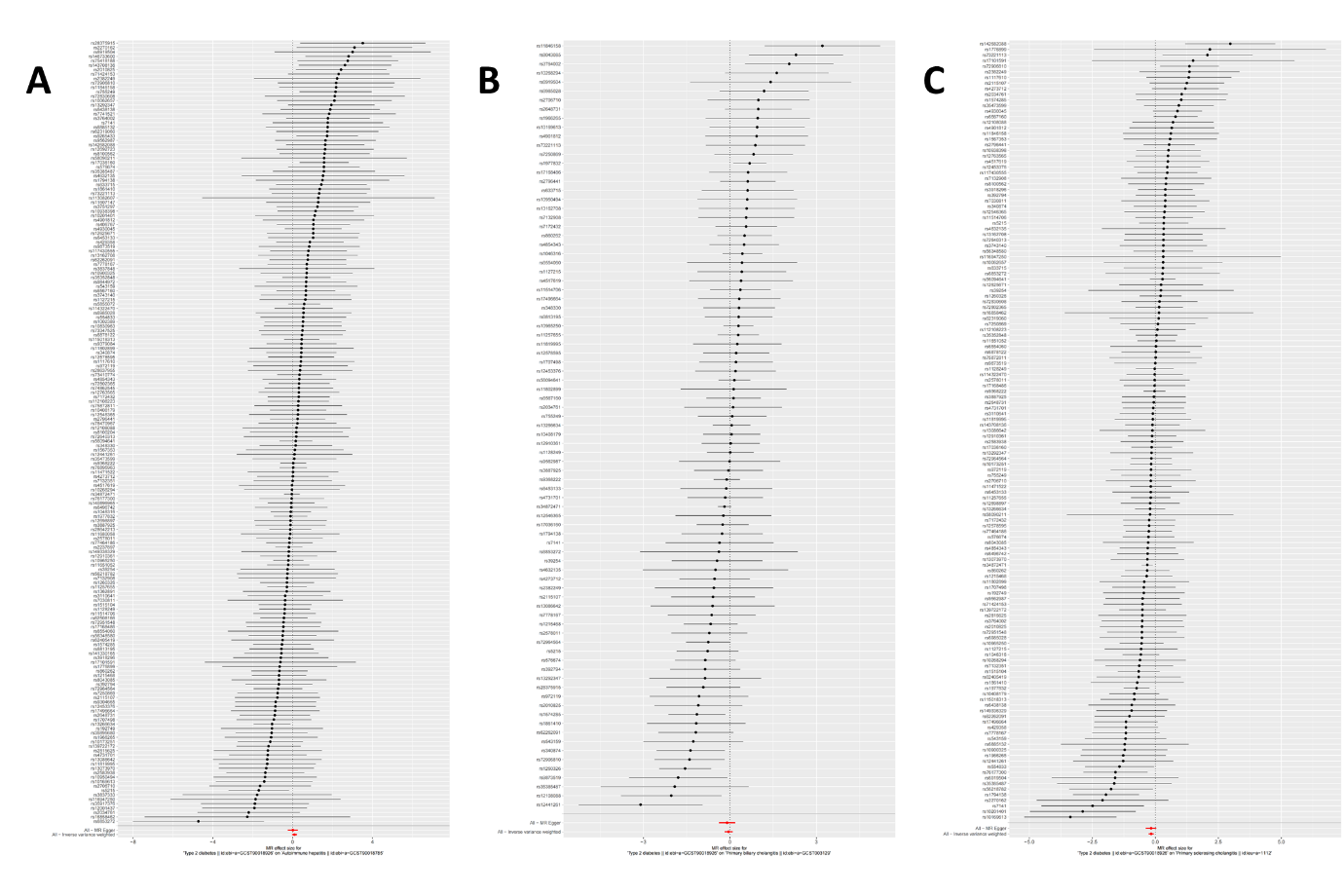


**Figure S4.** The funnel plot of reverse Mendelian randomization analysis. **(A)** T2D on AIH; **(B)** T2D on PBC; **(C)** T2D on PSC. T2D, type 2 diabetes; AIH, autoimmune hepatitis; PBC, primary biliary cholangitis; PSC, primary sclerosing cholangitis.


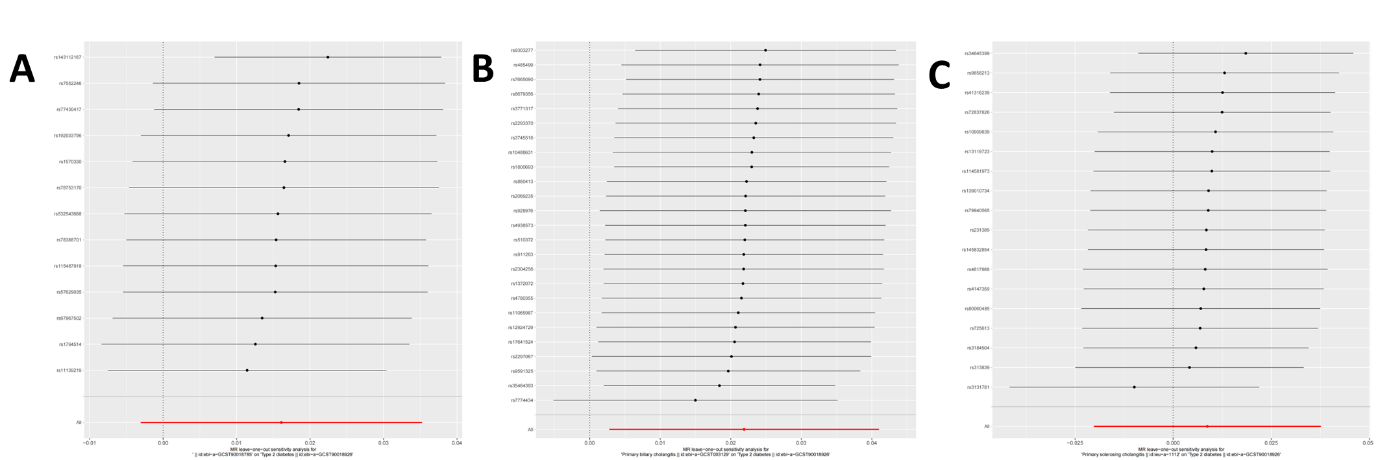


**Figure S5.** The forest plot of reverse Mendelian randomization analysis. **(A)** AIH on T2D; **(B)** PBC on T2D; and **(C)** PSC on T2D. AIH, autoimmune hepatitis; PBC, primary biliary cholangitis; PSC, primary sclerosing cholangitis; T2D, type 2 diabetes.


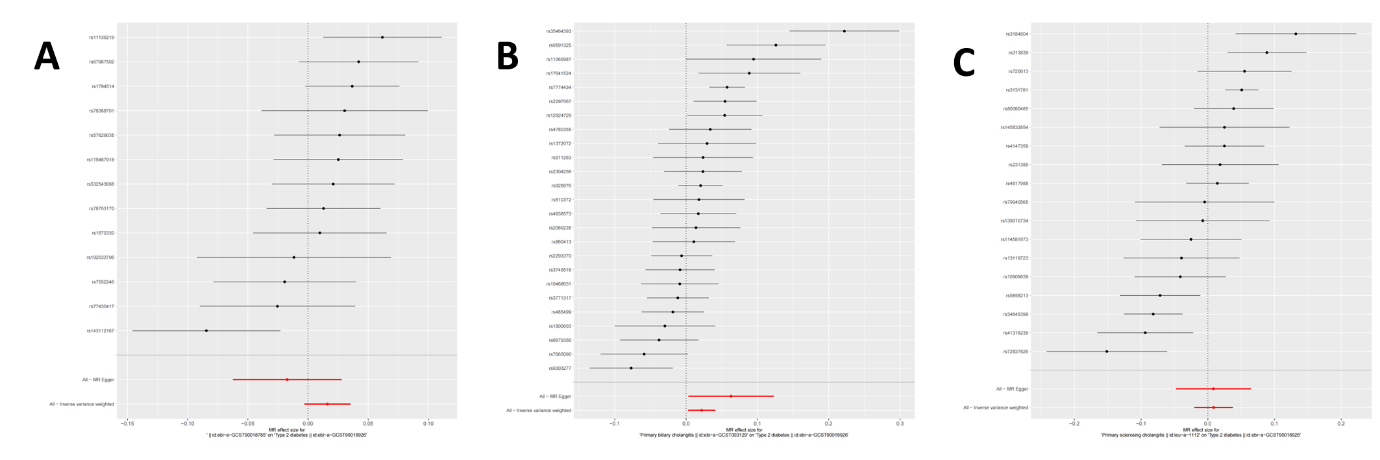


**Tables S1. STROBE-MR checklist of recommended items to address in reports of Mendelian randomization studies**

| **Item No.** | **Section** | **Checklist item** | **Page No.** | **Relevant text from manuscript** |
| --- | --- | --- | --- | --- |
| 1 | **TITLE and ABSTRACT** | Indicate Mendelian randomization (MR) as the study’s design in the title and/or the abstract if that is a main purpose of the study | 1 | Association between type 2 diabetes and autoimmune liver disease: an integrated analysis of mendelian randomization and clinical samples |
|  | **INTRODUCTION** |  |  |  |
| 2 | **Background** | Explain the scientific background and rationale for the reported study. What is the exposure? Is a potential causal relationship between exposure and outcome plausible? Justify why MR is a helpful method to address the study question | 3, 4 | In recent years, several observational studies have emphasized the link between T2D and AILD. A propensity score-matched analysis applying United Kingdom Biobank data showed that AILD was associated with a high incidence of T2D. It is well known that the liver plays an important role in maintaining glucose homeostasis in the body. Immunogenic liver injury may affect the body's glucose metabolism as well as insulin secretion levels. In addition, a large observational study showed that T2D is associated with an increased risk of hospital admission or death for AILD. However, it is important to note that the results of the observational studies mentioned may be influenced by various confounding variables, such as obesity and inflammatory bowel disease. Additionally, the possibility of reverse causality cannot be ruled out. Therefore, it is crucial to use higher precision experimental methods to clarify potential causal associations between T2D and AILD.  Mendelian randomization (MR) is an epidemiological approach based on genome-wide association study (GWAS) data to infer potential causal relationships between risk factor and outcomes. Because the alleles are randomly distributed during inheritance, analogous to randomized controlled trials (RCT), and occur during conception, MR minimizes the likelihood of confounding factors and reverse causality. Furthermore, the degree of reverse causal association between T2D and AILD was further validated through the collection and analysis of clinical samples, providing more reliable evidence for causal inference. |
| 3 | **Objectives** | State specific objectives clearly, including pre-specified causal hypotheses (if any). State that MR is a method that, under specific assumptions, intends to estimate causal effects | 4 | In the current study, we examine the causal relationship between T2D and AILD using MR analysis and clinical evidence. |
|  | **METHODS** |  |  |  |
| 4 | **Study design and data sources** | Present key elements of the study design early in the article. Consider including a table listing sources of data for all phases of the study. For each data source contributing to the analysis, describe the following: |  |  |
|  | a) | Setting: Describe the study design and the underlying population, if possible. Describe the setting, locations, and relevant dates, including periods of recruitment, exposure, follow-up, and data collection, when available. | 4 | The study design was conducted using a two-sample MR analysis. Specifically, we first delineated T2D as an exposure factor to perform forward MR analysis. Subsequently, we selected AILD as an exposure factor for a reverse MR analysis. |
|  | b) | Participants: Give the eligibility criteria, and the sources and methods of selection of participants. Report the sample size, and whether any power or sample size calculations were carried out prior to the main analysis | 5 | All GWAS data for exposures and endings were obtained from the Integrative Epidemiology Unit (IEU) Open GWAS project (<https://gwas.mrcieu.ac.uk/>), which is publicly available and does not require specific permissions for access. We screened the IVs for T2D from the recent publication's GWAS summary statistics, which including 38,841 T2D cases and 451,248 controls of European ancestry (GWAS ID: ebi-a-GCST90018926). The GWAS IDs for AIH (821 AIH cases and 484,413 controls of European ancestry) and PBC (2,764 PBC cases and 10,475 controls of European ancestry) were ebi-a-GCST90018785 and ebi-a-GCST003129, respectively. The GWAS summary statistics for PSC (GWAS ID: ieu-a-1112) included 2,871 PSC cases and 12,019 controls of European ancestry. Since the GWAS summary statistics were all publicly available, we did not require patient consent or informed consent. The details of these datasets in this study are presented in Table 1. |
|  | c) | Describe measurement, quality control and selection of genetic variants | 6 | We set strict quality control for the selection of IVs based on the three key hypotheses outlined by Bownden et al.. For the first hypothesis, the Single Nucleotide Polymorphisms (SNPs) closely associated with exposure variables were screened according to the p < 5×10-8 criterion. Due to the low number of AIH-associated IVs screened and to better comply with the random control principle, we used a more lenient threshold (p < 5 × 10-6) in our study based on previous experience. Subsequently, to keep SNPs independent, we performed linkage disequilibrium (LD) analysis (r2 > 0.001; clumping window < 10,000 kb) based on the European 1,000 Genomes Project reference panel. |
|  | d) | For each exposure, outcome, and other relevant variables, describe methods of assessment and diagnostic criteria for diseases | 8 | T2D diagnosis was based on diagnostic fasting glucose (≥ 7mmol/L), hemoglobin A1c levels (≥ 6.5%) and hospital discharge diagnosis.  All comply with the criteria set by the European Association for the Study of the Liver (EASL). |
|  | e) | Provide details of ethics committee approval and participant informed consent, if relevant | 7 | Ethical approval for the original study was obtained for all patients participating in the study. Since the GWAS summary statistics were all publicly available, therefore, we did not require patient consent or informed consent. |
| 5 | **Assumptions** | Explicitly state the three core IV assumptions for the main analysis (relevance, independence and exclusion restriction) as well assumptions for any additional or sensitivity analysis | 6 | In addition, it is worth noting that all research on MR analysis should adhere to the following three strict assumptions: (1) The chosen instrumental variables (IVs) must be guaranteed to be strongly correlated with the exposure phenotype, (2) IVs must be independent of confounding factors related to exposure phenotype and outcome, and (3) IVs can only affect the outcome through exposure phenotype, rather than through any other pathway. The study design is shown in Figure 1. |
| 6 | **Statistical methods: main analysis** | Describe statistical methods and statistics used |  |  |
|  | a) | Describe how quantitative variables were handled in the analyses (i.e., scale, units, model) |  | This research does not involve any transformations of quantitative variables. |
|  | b) | Describe how genetic variants were handled in the analyses and, if applicable, how their weights were selected | 7 | The inverse-variance weighted (IVW) method was applied as the main method of MR analysis to make a rigorous determination of the association between exposure and outcome. The main design idea of IVW is that, first, the Wald ratio is estimated individually for each SNP. Subsequently, a pooled effect size is obtained by aggregating the Wald ratios of all SNPs. Since its calculation process does not take into account the presence of an intercept term, IVW has the most accurate statistical results when there is no horizontal pleiotropy. |
|  | c) | Describe the MR estimator (e.g. two-stage least squares, Wald ratio) and related statistics. Detail the included covariates and, in case of two-sample MR, whether the same covariate set was used for adjustment in the two samples | 7 | The inverse-variance weighted (IVW) method was applied as the main method of MR analysis to make a rigorous determination of the association between exposure and outcome. In addition, we employed MR-Egger regression, weighted median method, simple mode, and weighted mode methods as additional analytical methods. When horizontal pleiotropy exists, MR-Egger regression can optimize the IVW estimate by modulating the intercept and regression slope. The weighted median method allows the results to remain stable when no more than 50% of the IVs are invalid. Finally, weighted mode method and simple mode method were used to further evaluate the credibility of IVW results. |
|  | d) | Explain how missing data were addressed |  | The issue of missing data was not involved. |
|  | e) | If applicable, indicate how multiple testing was addressed |  | In this MR analysis, multiple exposures or multiple outcomes were not involved. |
| 7 | **Assessment of assumptions** | Describe any methods or prior knowledge used to assess the assumptions or justify their validity | 7 | In addition, by calculating the F-statistic, we determined the probability that each SNP was a weak instrument. Based on previous experience, when F-statistic > 10, it indicates that there is no weak genetic instrument bias. The specific equation for the F-statistic is defined as: F = β^2^ / SE^2^, where β represents the effect size on exposure, and SE signifies the estimated standard error of SNP on exposure. Finally, palindromic alleles and incompatible alleles should be excluded. |
| 8 | **Sensitivity analyses and additional analyses** | Describe any sensitivity analyses or additional analyses performed (e.g. comparison of effect estimates from different approaches, independent replication, bias analytic techniques, validation of instruments, simulations) | 7, 8 | To confirm the robustness and reliability of the results, we performed a sensitivity analysis of the MR analysis. Specifically, potential heterogeneity was detected by Cochran's Q statistic. Where P > 0.05 indicates that there is no potential heterogeneity in the results, which was calculated using a fixed effects IVW model; otherwise, a random effects IVW model was chosen. Finally, the heterogeneity results were visualized by plotting a funnel plot. Furthermore, we used MR Egger regression to identify potential horizontal pleiotropy. The P-value of the MR-Egger intercept > 0.05 indicates that there is no horizontal pleiotropy. Finally, we assessed the impact of single SNPs on the overall results. |
| 9 | **Software and pre-registration** |  |  |  |
|  | a) | Name statistical software and package(s), including version and settings used | 10 | All statistical analyses were performed using the “TwoSampleMR” package (version 0.5.8) with R (version 4.2.3). |
|  | b) | State whether the study protocol and details were pre-registered (as well as when and where) |  | This study was not pre-registered with the study protocol and details. |
|  | **RESULTS** |  |  |  |
| 10 | **Descriptive data** |  |  |  |
|  | a) | Report the numbers of individuals at each stage of included studies and reasons for exclusion. Consider use of a flow diagram |  | This study provides the number of sample populations in the methodology section and does not provide information on the populations in the results. |
|  | b) | Report summary statistics for phenotypic exposure(s), outcome(s), and other relevant variables (e.g. means, SDs, proportions) |  | Summary data on exposure and outcomes are shown in Figure 2 and Figure 3. |
|  | c) | If the data sources include meta-analyses of previous studies, provide the assessments of heterogeneity across these studies |  | Detailed information is reported in Supplementary Table S2-S7 of the example text, e.g. the locus where each SNP is located, its size, the number of samples from which it originates and the size of the heterogeneity is reported. |
|  | d) | For two-sample MR:  i.  Provide justification of the similarity of the genetic variant-exposure associations between the exposure and outcome samples  ii.  Provide information on the number of individuals who overlap between the exposure and outcome studies |  | The data presented in this study were derived exclusively from European population samples. These samples were obtained from independent GWAS databases, ensuring minimal overlap and bias, detailed data on the number of individuals in the exposure and outcome samples are provided in Table 1. |
| 11 | **Main results** |  |  |  |
|  | a) | Report the associations between genetic variant and exposure, and between genetic variant and outcome, preferably on an interpretable scale |  | The example text reports the content of the entry in Supplementary Table S2-S7 of the Annex, including the number of instrumental variable SNPs, sample size, correlation between exposure-instrumental variables, and statistical validity. |
|  | b) | Report MR estimates of the relationship between exposure and outcome, and the measures of uncertainty from the MR analysis, on an interpretable scale, such as odds ratio or relative risk per SD difference | 10-12 | Based on the IVW method estimates, genetically predicted T2D was associated with a lower risk of PSC (OR = 0.847, 95% CI, 0.767–0.936, p = 0.001), and this result was supported by weighted median (OR = 0.826, 95% CI, 0.708–0.964, p = 0.016) and weighted mode (OR = 0.802, 95% CI, 0.661–0.972, p = 0.026). However, there was no evidence for a direct causal effect of T2D on AIH (IVW: OR = 1.093, 95% CI, 0.967–1.235, p = 0.153) and PBC (IVW: OR = 0.954, 95% CI, 0.829–1.098, p = 0.511).  To complete the directionality of the causal relationship between T2D and AILD, we also performed a reverse MR analysis. The IVW analysis results revealed that genetic prediction PBC had a significant correlation with the higher risk of T2D (OR = 1.022, 95% CI, 1.003–1.042, p = 0.025), and weighted median also showed similar results (OR = 1.019, 95% CI, 1.001–1.038, p = 0.035). However, the causal relationship between other types of AILD and T2D was insignificant, including AIH (IVW: OR = 1.093, 95% CI, 0.967–1.235, p = 0.153) and PSC (IVW: OR = 1.093, 95% CI, 0.967–1.235, p = 0.153). |
|  | c) | If relevant, consider translating estimates of relative risk into absolute risk for a meaningful time period |  | The calculation of absolute risk is detailed in Figure 2 and Figure 4. |
|  | d) | Consider plots to visualize results (e.g. forest plot, scatterplot of associations between genetic variants and outcome versus between genetic variants and exposure) |  | The results are visualized in Figure 3 and Supplementary Figure S4. |
| 12 | **Assessment of assumptions** |  |  |  |
|  | a) | Report the assessment of the validity of the assumptions | 10 | SNPs strongly associated with exposure were screened according to the screening criteria described above. Then, palindromic alleles and incompatible alleles were excluded after harmonization of exposure and outcome. Finally, there were 177 SNPs associated with T2D and AIH, 85 SNPs associated with T2D and PBC, 139 SNPs associated with T2D and PSC, 13 SNPs associated with AIH and T2D, 25 SNPs associated with PBC and T2D, and 18 SNPs associated with PSC and T2D that were identified, respectively. The F-values for each of the SNP is greater than 10, indicating that there was no weak IV. |
|  | b) | Report any additional statistics (e.g., assessments of heterogeneity across genetic variants, such as *I^2^*, Q statistic or E-value) |  | The additional statistics are provided in Table 2 and Table 3. |
| 13 | **Sensitivity analyses and additional analyses** |  |  |  |
|  | a) | Report any sensitivity analyses to assess the robustness of the main results to violations of the assumptions | 11, 12 | Cochran's Q test showed no heterogeneity in the results (T2D-AIH, p = 0.479; T2D-PSC, p = 0.088) except for T2D and PBC (p < 0.001). Therefore, IVW for T2D and PBC was modeled using a multiplicative random effects model. At the same time, we applied funnel plots to visualize the results of heterogeneity [Supplementary Figure S1 (A-C)]. In addition, the data from the Egger regression tests showed that no horizontal pleiotropy was observed for any of the results (T2D-AIH, p = 0.443; T2D-PBC, p = 0.668; T2D-PSC, p = 0.963), indicating that the results were not affected by potential confounders.  The results of Cochran's Q test revealed that there was no significant heterogeneity between AIH and T2D, and a fixed effects IVW model was used. We applied funnel plots to visualize the results of heterogeneity, as detailed in [Supplementary Figure S1 (D-F)]. Additionally, Egger regression tests did not detected any evidence of horizontal pleiotropy. |
|  | b) | Report results from other sensitivity analyses or additional analyses |  | The leave-one-out sensitivity analysis showed that the causal relationship between T2D and AILD was not affected by any single SNP, and thus our results are robust (Supplementary Figure S2 and Supplementary Figure S3). |
|  | c) | Report any assessment of direction of causal relationship (e.g., bidirectional MR) |  | The results showed that T2D is associated with a reduced risk of PSC. In addition, there is a positive genetic association between PBC and an increased risk of T2D. However, no potential causal relationships were observed between T2D and AIH, T2D and PBC, AIH and T2D, and PSC and T2D. |
|  | d) | When relevant, report and compare with estimates from non-MR analyses | 13, 14 | Data from the clinical sample indicated that a total of 24 out of 87 AILD patients (27.6%) had T2D. Among these, 9 AIH patients (23.7%) and 15 PBC patients (30.6%) were diagnosed with T2D. Subsequently, we categorized patients with each subtype of AILD based on the presence or absence of comorbid T2D and analyzed their baseline data (Table 4). The results indicated that the prevalence of hypertension was significantly higher in patients with AIH who also had T2D compared to those without T2D (p = 0.016). Furthermore, among patients with PBC, those with T2D exhibited higher IgG test values (p = 0.018) and lower total bilirubin levels (p = 0.046) than patients without T2D. These differences were statistically significant. Multifactorial logistic regression analysis revealed that elevated IgG levels were an independent risk factor for the development of T2D in patients with PBC (OR = 11.30, 95% CI, 1.02-124.89, p = 0.048). Additionally, high total bilirubin levels were identified as a protective factor that reduced the likelihood of developing T2D in patients with PBC (OR = 0.02, 95% CI, 0.001-0.33, p = 0.006). Unfortunately, no independent influencing factors for the occurrence of combined T2D in patients with AIH were identified (Table 5). |
|  | e) | Consider additional plots to visualize results (e.g., leave-one-out analyses) |  | Visualisation pictures of all results are shown in Figure 3 and Supplementary Figure S1-S5. |
|  | **DISCUSSION** |  |  |  |
| 14 | **Key results** | Summarize key results with reference to study objectives | 14 | The results showed that T2D is associated with a reduced risk of PSC. In addition, there is a positive genetic association between PBC and an increased risk of T2D. However, no potential causal relationships were observed between T2D and AIH, T2D and PBC, AIH and T2D, and PSC and T2D. |
| 15 | **Limitations** | Discuss limitations of the study, taking into account the validity of the IV assumptions, other sources of potential bias, and imprecision. Discuss both direction and magnitude of any potential bias and any efforts to address them | 18 | However, a full discussion of the limitations of this study is necessary. First, we evaluated the results using strict thresholds and other test hypotheses, which may produce falsely positive results. Second, because the GWAS data did not provide detailed information on patients with each disease, a more detailed subgroup analysis could not be performed to elucidate the specificity of the results. Third, the genetic background of this study exclusively comprised individuals of European descent. Therefore, the generalizability of our findings to populations worldwide might be limited. Additionally, our clinical data were constrained by the low prevalence of the disease and the single-center design of the study, which results in a relatively small sample size and a lack of control groups. Finally, this clinical study did not validate a forward MR analysis, which requires further investigation in larger prospective studies. |
| 16 | **Interpretation** |  |  |  |
|  | a) | Meaning: Give a cautious overall interpretation of results in the context of their limitations and in comparison with other studies |  | The present study hints at a causal relationship between T2D and AILD through genetic means. |
|  | b) | Mechanism: Discuss underlying biological mechanisms that could drive a potential causal relationship between the investigated exposure and the outcome, and whether the gene-environment equivalence assumption is reasonable. Use causal language carefully, clarifying that IV estimates may provide causal effects only under certain assumptions | 16, 17 | Notably, the liver is key in regulating glucose homeostasis, which is disrupted in vivo when immunogenic liver injury occurs. At the same time, activated immune factors increase collagen-I levels through hepatic stellate cells (HSC) autophagy, which promotes hepatic fibrosis and thus triggers insulin resistance. Accelerated the occurrence and development of T2D. Coincidentally, in 2019, a Belgian case report reported that the application of desiccated ursodeoxycholic acid significantly alleviated the level of hepatic fibrosis in patients with PBC and simultaneously improved insulin resistance. In addition, it has been established that bile acids play an important role in glucose homeostasis. Intrahepatic cholestasis is a characteristic manifestation of PBC and may result in a severe disorder of bile acid circulation. Stagnant bile acids disrupt glucose metabolism by mediating multiple signaling pathways, thereby increasing the risk of developing T2D. Despite the existence of multiple potential biological mechanisms, the limited research on the association between PSC and T2D requires further investigation. |
|  | c) | Clinical relevance: Discuss whether the results have clinical or public policy relevance, and to what extent they inform effect sizes of possible interventions | 18 | The present study provided new insights into the clinical practice for patients with T2D and AILD. |
| 17 | **Generalizability** | Discuss the generalizability of the study results (a) to other populations, (b) across other exposure periods/timings, and (c) across other levels of exposure | 18 | The genetic background of this study exclusively comprised individuals of European descent. Therefore, the generalizability of our findings to populations worldwide might be limited. |
|  | **OTHER INFORMATION** |  |  |  |
| 18 | **Funding** | Describe sources of funding and the role of funders in the present study and, if applicable, sources of funding for the databases and original study or studies on which the present study is based | 20 | This research was supported by the Hebei Provincial Key Research and Development Program Project (21377767D). |
| 19 | **Data and data sharing** | Provide the data used to perform all analyses or report where and how the data can be accessed, and reference these sources in the article. Provide the statistical code needed to reproduce the results in the article, or report whether the code is publicly accessible and if so, where | 20 | Details are provided in the data availability statement and supplementary materials. |
| 20 | **Conflicts of Interest** | All authors should declare all potential conflicts of interest | 20 | The authors declare that the research wasno competing interests. |

This checklist is copyrighted by the Equator Network under the Creative Commons Attribution 3.0 Unported (CC BY 3.0) license.

1. Skrivankova VW, Richmond RC, Woolf BAR, Yarmolinsky J, Davies NM, Swanson SA, et al. Strengthening the Reporting of Observational Studies in Epidemiology using Mendelian Randomization (STROBE-MR) Statement. JAMA. 2021;under review.

2. Skrivankova VW, Richmond RC, Woolf BAR, Davies NM, Swanson SA, VanderWeele TJ, et al. Strengthening the Reporting of Observational Studies in Epidemiology using Mendelian Randomisation (STROBE-MR): Explanation and Elaboration. BMJ. 2021;375:n2233.
